# Reproductive, Dietary, and Physical Activity Factors Associated With Multiple Sclerosis: A Case–Control Study

**DOI:** 10.1002/hsr2.72773

**Published:** 2026-07-01

**Authors:** Nazanin Razazian, Shiva Bashiri, Asma Aliahmadi, Sharareh Eskandarieh, Mohammad Ali Sahraian, Milad MohamadYari, Mansour Rezaei, Negin Fakhri

**Affiliations:** ^1^ Neuroscience Research Center, Health Institute Kermanshah University of Medical Sciences Kermanshah Iran; ^2^ Clinical Research Development Center, Imam Reza Hospital Kermanshah University of Medical Sciences Kermanshah Iran; ^3^ Multiple Sclerosis Research Center, Neuroscience Institute Tehran University of Medical Sciences Tehran Iran; ^4^ Social Development and Health Promotion Research Center Kermanshah University of Medical Sciences Kermanshah Iran

**Keywords:** Environmental Risk Factors, Lifestyle Determinants, Multiple Sclerosis, Nutrition, Physical Activity, Reproductive Health

## Abstract

**Background and Aims:**

Multiple sclerosis (MS) is a chronic inflammatory disease influenced by genetic and environmental factors. Dietary habits, reproductive characteristics, and physical activity have been proposed as potential contributors to MS susceptibility. This study aimed to investigate the association of dietary intake, supplement use, reproductive factors, and physical activity with MS in Kermanshah Province, Iran.

**Methods:**

This case–control study included 600 participants (300 MS patients and 300 matched healthy controls) from Kermanshah. Data were collected using the validated Persian version of the Environmental Exposure and Lifestyle Risk Factors in MS Questionnaire (EnvIMS‐Q). Logistic regression analyses were performed to estimate crude and adjusted odds ratios (ORs) with 95% confidence intervals (CIs).

**Results:**

Compared with controls, participants with MS reported lower consumption of several foods, including fish (adjusted OR = 0.31, 95% CI 0.19–0.50), seafood (adjusted OR = 0.31, 95% CI 0.10–0.98), nuts (adjusted OR = 0.60, 95% CI 0.39–0.93), and dairy products. Fish oil supplementation was less common among patients with MS (adjusted OR = 0.28, 95% CI 0.13–0.61). Female patients reported higher frequencies of oral contraceptive use, miscarriage, and assisted reproduction. Vigorous physical activity was significantly lower among patients than controls, whereas mild physical activity did not differ significantly. No significant association was observed for vitamin D supplementation.

**Conclusion:**

This study identified associations between several dietary, reproductive, and lifestyle factors and MS in a high‐prevalence region of Iran. Lower consumption of selected nutrient‐rich foods, lower fish oil supplement use, certain reproductive factors, and lower levels of vigorous physical activity were associated with higher odds of MS. Prospective studies are needed to clarify temporal relationships and causality.

## 1 Introduction

Multiple sclerosis (MS) is a chronic inflammatory and immune‐mediated disease of the central nervous system (CNS), characterized by demyelination, axonal damage, and progressive neurodegeneration. It is one of the most common non‐traumatic causes of disability among young adults, with a rising global prevalence that imposes substantial medical, social, and economic burdens [1, 2]. Clinically, MS presents in several forms, including relapsing‐remitting MS (RRMS), secondary progressive MS (SPMS), primary progressive MS (PPMS), and the less frequent progressive‐relapsing MS (PRMS), with RRMS accounting for approximately 85% of cases [3].

Epidemiological evidence highlights the heterogeneous distribution of MS worldwide, ranging from less than 5 per 100,000 in tropical regions to more than 200 per 100,000 in parts of North America and Europe [2, 4]. In recent decades, Iran has reported a sharp increase in MS prevalence, with the province of Kermanshah being among the high‐risk regions [5, 6, 7]. This trend has raised growing concerns regarding disease burden and the urgent need for preventive strategies.

Although the precise etiology of MS remains unclear, it is widely accepted that a complex interplay of genetic susceptibility and environmental exposures contributes to disease onset [8, 9, 10, 11]. Among the environmental factors, nutrition has been increasingly emphasized. Diets rich in antioxidants, vitamin D, and polyunsaturated fatty acids have been associated with neuroprotection and reduced inflammation, while vitamin D deficiency in particular is consistently linked to both disease risk and progression [11, 12].

Hormonal factors are also significant in explaining the sex‐related differences in MS prevalence [13]. Estrogens modulate immune responses and may influence disease course. For example, the use of oral contraceptives has been associated with a reduced risk of MS, whereas pregnancy and postpartum conditions are known to alter disease activity [14].

Physical activity, meanwhile, is considered a potential protective factor. Regular exercise has been shown to improve aerobic capacity, muscular strength, and health‐related quality of life in MS patients, while also reducing systemic inflammation and modulating immune function. Importantly, prospective studies suggest that higher levels of physical activity may lower both the risk of developing MS and its subsequent progression [15].

Given the high prevalence of MS in Iran—particularly in Kermanshah province—and the growing body of evidence linking lifestyle‐related risk factors such as diet, hormonal status, and physical activity with disease susceptibility, a deeper investigation into these associations is crucial. Understanding these relationships can not only enhance preventive strategies but also guide public health interventions and inform individualized approaches to disease management.

## 2 Materials and Methods

### 2.1 Study Design and Participants

The study was conducted in Kermanshah province, Iran, from July 2024 to October 2025. A total of 600 participants were recruited, consisting of 300 patients with confirmed MS and 300 healthy controls. All MS patients were diagnosed by neurologists based on clinical history, neurological examination, paraclinical investigations, and the 2017 McDonald criteria [16]. Patients with MS were identified from the National Multiple Sclerosis Registry of Iran (NMSRI) [17], Kermanshah. The sample size was determined by the availability of eligible participants during the study period, and all individuals meeting the inclusion criteria were invited to participate.

### 2.2 Inclusion and Exclusion Criteria

Inclusion criteria for cases were: confirmed MS diagnosis, age between 18 and 50 years, residency in Kermanshah province for at least 2 years, and willingness to participate.

Exclusion criteria for cases included: presence of other neurological disorders, severe head trauma with loss of consciousness, or chronic comorbid diseases.

Inclusion criteria for controls were: age between 18 and 50 years, no history of MS or neurological disease, residency in Kermanshah for at least 2 years, and consent to participate.

Exclusion criteria for controls included: pregnancy, history of neurological disease, major chronic illness, or inability to provide informed consent.

### 2.3 Sampling and Data Collection

Participants were recruited through convenience sampling. Patients with MS were identified through the Kermanshah branch of NMSRI and were contacted through Imam Reza Hospital. Healthy controls were recruited from the same underlying source population as the cases, namely residents of Kermanshah Province without a history of neurological or autoimmune diseases. Healthy volunteers were recruited from community settings as well as companions or relatives of non‐neurological patients attending Imam Reza Hospital. Controls were frequency‐matched to cases according to age and sex. Demographic and clinical data for patients were collected from the Kermanshah provincial NMSRI and medical records. After initial screening, participants were invited for face‐to‐face interviews, during which the study aims were explained and written informed consent was obtained. For both cases and controls, data were collected using the Environmental Exposure and Lifestyle Risk Factors in Multiple Sclerosis Questionnaire (EnvIMS‐Q), which has been validated and culturally adapted into Persian with established reliability [18]. Interviews were conducted by two trained neurology residents who had received standardized instruction on questionnaire administration to ensure consistency and accuracy. To minimize interviewer and recall bias, the same standardized questionnaire and interview protocol were used for both groups by the same trained interviewers. All interviews were performed in a private setting to maintain confidentiality. Questionnaires were anonymized and coded for data security.

### 2.4 Variables and Measurements

The questionnaire covered a wide range of demographic, lifestyle, nutritional, hormonal, and physical activity factors [13, 19]. Nutritional intake was assessed by frequency of consumption of major food groups (dairy products, fish, meat, fruits, vegetables, nuts, fast food, and supplements). Dietary variables reflected participants’ self‐reported usual weekly consumption patterns [13]. Hormonal factors included contraceptive use, pregnancy history, and fertility treatments [19]. Reproductive variables were collected as self‐reported categorical variables. Physical activity was measured in terms of frequency, duration, and intensity of mild and vigorous activities [20]. Supplement use was recorded as a yes/no exposure.

### 2.5 Statistical Analysis

Statistical analyses were performed using the Statistical Package for the Social Sciences (SPSS) version 29.0 (IBM Corp., Armonk, NY, USA). Continuous variables were summarized as mean ± standard deviation (SD), and categorical variables as frequencies and percentages. Normality of continuous variables was assessed prior to analysis.

Between‐group comparisons were conducted using the independent‐samples *t*‐test or Mann–Whitney *U* test for continuous variables, as appropriate, and the chi‐square test for categorical variables. For categorical variables with small expected cell counts, Fisher's exact test was used when appropriate.

Binary logistic regression analyses were performed to estimate crude and adjusted odds ratios (ORs) with corresponding 95% confidence intervals (CIs) for factors associated with MS. Sex was included as an adjustment variable in multivariable models because of the observed imbalance between groups. Results are presented as effect estimates together with their 95% confidence intervals to facilitate interpretation of both the magnitude and precision of associations.

All analyses were pre‐specified according to the study objectives. All statistical tests were two‐sided, and a *p* value < 0.05 was considered statistically significant.

### 2.6 Ethical Considerations

This study was approved by the Ethics Committee of Kermanshah University of Medical Sciences (Ethics Code: IR.KUMS.MED.REC.1403.059). Written informed consent was obtained from all participants prior to enrollment. All collected data were anonymized, coded, and analyzed confidentially to ensure the privacy.

## 3 Results

All enrolled participants were included in the final analyses, and no participants were excluded after recruitment. A total of 600 participants were enrolled in the study, comprising 300 patients with MS and 300 healthy controls. Although controls were frequency‐matched to cases by age and sex, a modest imbalance in sex distribution remained between groups. Women represented the majority in both groups, but the proportion was higher among MS patients (78.1% vs. 70.7%, *p* = 0.03); Therefore, sex was included as an adjustment variable in all multivariable logistic regression models.

### 3.1 Reproductive and Hormonal Factors

Among female participants, oral contraceptive pill (OCP) use was more common among women with MS (OR 1.50, 95% CI 1.03–2.19; *p* = 0.03). Miscarriage was associated with higher odds of MS (OR 1.71, 95% CI 1.04–2.83; *p* = 0.04). Assisted reproduction was more frequent among MS patients (OR 4.14, 95% CI 1.17–14.63; *p* = 0.03). No significant differences were found regarding menstrual regularity or breastfeeding history. (Table 1).

**Table 1:** Demographic, socioeconomic, and reproductive characteristics of participants.

Variable	MS patients (*n* = 300)	Controls (*n* = 300)	OR (95% Cl)	*p*‐value
Sex (female), *n* (%)	232(78.1%)	212 (70.7%)	—	0.03
Age, mean ± SD	37.05 ± 7.37	36.13 ± 9.09	—	0.36
Married, *n* (%)	191 (64.3%)	196 (65.3%)	—	0.79
OCP use, *n* (%)*	115 (49.1%)	83 (39.2%)	1.50 (1.03, 2.19)	0.03
Regular menstruation, *n* (%)*	168(71.8%)	154 (76.6%)	0.78 (0.48, 1.25)	0.30
Miscarriage, *n* (%)*	54 (23.1%)	29 (14.4%)	1.71 (1.04, 2.83)	0.04
Assisted reproduction (IUI/IVF), *n* (%)*	14 (6.0%)	3 (1.5%)	4.14 (1.17, 14.63)	0.03
History of breastfeeding, *n* (%)*	111 (47.4%)	95 (47.3%)	1.12 (0.75, 1.67)	0.59

*Note:* ORs were obtained from univariate logistic regression. Reference category for binary variables = No exposure (e.g., No Miscarriage, No history of IUI/IVF, etc.).

Abbreviations: CI = Confidence Interval, IUI = Intra Uterine Insemination, IVF = In Vitro Fertilization, MS = Multiple Sclerosis, OCP = Oral Contraceptive Pills, OR = Odds Ratio, SD = Standard Deviation.

* Among women only.

### 3.2 Nutritional Factors

Dietary assessment revealed several differences between the groups (Table 2). Higher milk consumption was associated with lower odds of MS (aOR 0.67, 95% CI 0.46–0.97; *p* = 0.03). Higher yogurt consumption was associated with lower odds of MS (aOR 0.62, 95% CI 0.43–0.89; *p* = 0.01). Higher cheese consumption was associated with lower odds of MS (aOR 0.67, 95% CI 0.47–0.95; *p* = 0.03). Higher red meat consumption was associated with lower odds of MS (aOR 0.67, 95% CI 0.45–0.99; *p* = 0.04). Furthermore, Higher fish consumption was associated with substantially lower odds of MS (aOR 0.31, 95% CI 0.19–0.50; *p* < 0.001). Higher seafood consumption was associated with lower odds of MS (aOR 0.31, 95% CI 0.10–0.98; *p* = 0.045). Higher nut consumption was associated with lower odds of MS (aOR 0.60, 95% CI 0.39–0.93; *p* = 0.02). Pizza and burger consumption were reported more frequently among controls than among MS patients. (Table 2).

**Table 2:** Weekly food consumption in MS patients and controls.

Food item (serving/week)	MS patients (*n* = 300)	Controls (*n* = 300)	Crude OR (95% Cl)	*p*‐value	Adjusted OR (95% Cl)	*p*‐value
Milk	2.52 ± 2.17	2.76 ± 1.79	0.69 (0.48, 0.99)	0.05	0.67 (0.46, 0.97)	0.03
Yogurt	3.11 ± 2.26	3.42 ± 2.48	0.61 (0.43, 0.88)	0.008	0.62 (0.43, 0.89)	0.01
Cheese	4.06 ± 2.43	4.69 ± 2.60	0.70 (0.49, 0.99)	0.04	0.67 (0.47, 0.95)	0.02
Poultry (chicken/turkey)	2.86 ± 2.32	2.90 ± 1.42	0.82 (0.55, 1.22)	0.33	0.83 (0.56, 1.23)	0.36
Red meat	2.63 ± 1.46	3.03 ± 1.78	0.68 (0.46, 1.00)	0.05	0.67 (0.45, 0.99)	0.04
Eggs	3.22 ± 1.82	3.06 ± 1.63	1.23 (0.84, 1.80)	0.29	1.26 (0.85, 1.86)	0.25
Fish	0.18 ± 0.49	0.44 ± 0.76	0.33 (0.20, 0.54)	< 0.001	0.31 (0.19, 0.50)	< 0.001
Shrimp/Seafood	0.01 ± 0.12	0.06 ± 0.26	0.28 (0.09, 0.87)	0.03	0.31 (0.10, 0.98)	0.045
Fruits	4.75 ± 2.14	5.03 ± 2.23	0.84 (0.60, 1.18)	0.31	0.83 (0.59, 1.17)	0.29
Vegetables	3.02 ± 2.70	3.03 ± 1.87	0.70 (0.48, 1.03)	0.46	0.69 (0.48, 1.01)	0.36
Nuts	1.70 ± 2.00	2.06 ± 1.92	0.60 (0.39, 0.92)	0.02	0.60 (0.39, 0.93)	0.02
Sausage/processed meat	0.77 ± 0.99	0.78 ± 0.91	1.29 (0.83, 2.02)	0.26	1.27 (0.81, 1.99)	0.30
Pizza	0.44 ± 1.16	1.11 ± 1.94	0.33 (0.21, 0.54)	< 0.001	0.35 (0.22, 0.56)	< 0.001
Burger	0.48 ± 0.83	0.63 ± 0.77	0.58 (0.40, 0.84)	0.004	0.60 (0.41, 0.87)	0.008

*Note:* For exploratory analyses, continuous dietary variables were categorized according to their median values and entered into logistic regression models. Participants with values below the median were assigned to the reference category (coded as 0), and those with values at or above the median were coded as 1. Crude odds ratios (ORs) were estimated using univariate logistic regression analyses. Multivariable logistic regression models adjusted for sex were applied to obtain adjusted ORs.

Abbreviations: CI = Confidence Interval, MS = Multiple Sclerosis, OR = Odds Ratio.

### 3.3 Supplement Use

As shown in Table 3, Vitamin C supplementation was associated with lower odds of MS (aOR 0.63, 95% CI 0.44–0.91; *p* = 0.01). Fish oil supplementation was associated with markedly lower odds of MS (aOR 0.28, 95% CI 0.13–0.61; *p* = 0.001). No significant differences were observed for vitamin D, multivitamins, calcium, iron, folic acid, or vitamin B12.

**Table 3:** Supplement use among MS patients and controls.

Supplement	MS patients (*n* = 300)	Controls (*n* = 300)	Crude OR (95% Cl)	*p*‐value	Adjusted OR (95% Cl)	*p*‐value
Vitamin D supplements	58 (19.5%)	63 (21.7%)	0.80 (0.54, 1.21)	0.30	0.73 (0.49, 1.08)	0.12
Vitamin C supplements	74 (25.2%)	99 (33.9%)	0.62 (0.43, 0.89)	0.01	0.63 (0.44, 0.91)	0.01
Fish oil supplements	9 (3%)	28 (9.5%)	0.28 (0.13, 0.60)	0.001	0.28 (0.13, 0.61)	0.001
Multivitamins	75 (25.3%)	77(26.6%)	0.92 (0.63, 1.34)	0.66	0.92 (0.63, 1.35)	0.68
Calcium	53 (17.9%)	66(22.8%)	0.73 (0.48, 1.10)	0.13	0.73 (0.48, 1.10)	0.14
Iron	89 (30.2%)	87(29.8%)	0.95 (0.66, 1.36)	0.76	0.90 (0.62, 1.29)	0.56
Folic acid	64 (21.5%)	57(19.6%)	1.07 (0.71, 1.61)	0.73	1.01 (0.67, 1.53)	0.95
Vitamin B12	46 (15.5%)	58 (19.9%)	0.69 (0.45, 1.07)	0.10	0.69 (0.45, 1.06)	0.09

*Note:* Crude ORs were obtained from univariate logistic regression. Adjusted ORs were derived from multivariable models adjusted for sex.

Abbreviations: CI = Confidence Interval, MS = Multiple Sclerosis, OR = Odds Ratio.

### 3.4 Physical Activity

Physical activity levels differed significantly between groups (Table 4). While mild activity did not differ in terms of frequency or duration, vigorous activity was substantially lower among MS patients.

**Table 4:** Physical activity among MS patients and controls.

Physical activity measure	MS patients (*n* = 300)	Controls (*n* = 300)	*p*‐value
Mild activity (sessions/week)	4.36 ± 2.19	4.19 ± 1.88	0.27
Mild activity (min/session)	46.85 ± 46.63	47.12 ± 45.85	0.17
Vigorous activity (sessions/week)	2.13 ± 1.32	4.29 ± 1.29	< 0.001
Vigorous activity (min/session)	32.40 ± 32.41	38.37 ± 32.66	0.03

Abbreviations: Min = Minute, MS = Multiple Sclerosis.

## 4 Discussion

This study investigated dietary intake, supplement use, reproductive factors, and physical activity in relation to MS risk in Kermanshah. The results suggest associations between several modifiable exposures and MS in this high‐prevalence region.

Patients consumed significantly less milk, yogurt, cheese, red meat, nuts, fish, and seafood compared with controls, while pizza was consumed more frequently among controls. These findings indicate that lower intake of these foods was associated with higher odds of MS in this study population. Several studies have suggested that vitamin D, omega‐3 fatty acids, and antioxidants present in fish, dairy, and nuts, which may contribute to immune regulation and lower inflammatory activity [2, 11, 12, 21, 22]. For example, prospective studies have reported that higher fish intake is associated with lower MS risk, likely due to both vitamin D and marine fatty acids. Similarly, lower consumption of dairy products and red meat has been associated with lower intake of vitamin D, calcium, and iron, nutrients that may influence immune and neurological function [11, 12, 23]. Higher pizza consumption was observed among controls than among participants with MS. However, this finding should be interpreted cautiously, as the case–control design does not allow causal inference and dietary habits may have changed following disease diagnosis. Therefore, the observed difference may reflect post‐diagnosis dietary modifications rather than a direct association between pizza consumption and MS.

Although vitamin D deficiency is consistently reported as a major risk factor for MS [11, 12, 21, 22], our study did not find significant differences in supplementation between patients and controls. This may reflect the widespread supplementation programs in Iran. Nonetheless, the lower use of fish oil and vitamin C supplements among patients is consistent with evidence that omega‐3 fatty acids and antioxidants may reduce neuroinflammation and support myelin integrity [11, 12, 23].

Female patients had higher rates of OCP use, miscarriage, and assisted reproduction compared with controls. The relationship between OCPs and MS risk remains controversial: while some reports suggest a protective effect through hormonal modulation [14], others have shown no effect or even increased risk. Our findings add to the existing body of evidence, although the observed associations should be interpreted cautiously, given the observational nature of the study. Reproductive factors such as miscarriage and infertility treatments may indicate underlying immune dysregulation, consistent with prior studies linking reproductive health to MS risk [14, 24, 25]. Notably, some studies have reported that gonadotropin‐releasing hormone (GnRH) agonist therapy, commonly used during assisted reproductive procedures, may exacerbate MS disease activity, possibly through transient immune activation or hormonal fluctuations [26, 27, 28]. This observation aligns with our findings, supporting the hypothesis that hormonal manipulation in fertility treatments may influence disease course.

Patients reported lower levels of vigorous activity than controls, while mild activity did not differ significantly. These results are in line with evidence that regular vigorous physical activity has been associated with lower systemic inflammation, modulation of immune activity, and improved neurological outcomes [7, 15, 24]. Studies in Iran and abroad have shown that higher levels of exercise are associated with lower odds of MS and slower disease progression, supporting a potential association between higher physical activity levels and more favorable MS‐related outcomes [7, 15, 29].

An important consideration when interpreting the present findings is that several environmental and lifestyle exposures associated with MS may exert their effects long before the clinical onset of disease. Recent evidence suggests that childhood and adolescence represent critical periods during which environmental factors may influence future MS susceptibility. A recent systematic review and meta‐analysis reported that multiple pediatric environmental exposures are associated with later MS risk [30]. Therefore, some of the dietary and lifestyle associations observed in our study may reflect long‐term exposure patterns that began years before disease manifestation rather than only current adult behaviors.

## 5 Strengths and Limitations

This study has several strengths. It included a relatively large sample size with equal numbers of cases and controls, which provided adequate statistical power for comparisons. The groups were frequency‐matched for age and sex, and efforts were made to minimize selection bias through recruitment from a well‐defined population. Moreover, the study simultaneously assessed multiple domains of potential risk factors—including reproductive, nutritional, supplement use, and physical activity—which allowed for a more comprehensive evaluation of lifestyle and biological determinants of MS.

Nevertheless, some limitations should be considered. Despite frequency matching by age and sex, a statistically significant imbalance in sex distribution remained between groups. Although sex was included as an adjustment variable in multivariable analyses, residual confounding cannot be completely excluded. In addition, as a case–control design, causal relationships between exposures and MS cannot be established. Information on diet, supplement use, physical activity, and reproductive history was collected by self‐reported questionnaires, which may be subject to recall bias and misclassification. Furthermore, dietary exposures were assessed based on self‐reported weekly consumption patterns at the time of data collection. Information regarding the duration and long‐term stability of these dietary habits was unavailable; therefore, exposure misclassification cannot be excluded. In addition, other potential confounders such as genetic susceptibility, comorbid conditions, and environmental exposures were not assessed. Finally, several continuous exposure variables were categorized according to their median values because clinically established cut‐off points were unavailable; therefore, some loss of information cannot be excluded.

## 6 Conclusion

Our findings indicate that reduced consumption of fish, dairy, nuts, and red meat, along with lower use of protective supplements, reproductive risk factors, and reduced vigorous physical activity, are associated with higher odds of MS in Kermanshah. These results highlight the importance of diet and lifestyle modifications, reproductive health awareness, and exercise promotion as potentially important public health considerations in populations at elevated risk of MS.

## Author Contributions

**Nazanin Razazian:** project administration, writing – review and editing, supervision, funding acquisition. **Shiva Bashiri:** conceptualization, methodology, investigation, writing – original draft. **Asma Aliahmadi:** conceptualization, methodology, data curation, investigation. **Sharareh Eskandarieh:** conceptualization, methodology, validation, writing – review and editing. **Mohammad Ali Sahraian:** conceptualization, methodology, supervision, validation. **Milad Mohamad Yari:** writing – original draft, writing – review and editing, formal analysis, software. **Mansour Rezaei:** methodology, data curation, software, visualization. **Negin Fakhri:** software, methodology, resources.

## Conflicts of Interest

The authors declared no conflicts of interest.

## Transparency Statement

Milad MohamadYari affirms that this manuscript is an honest, accurate, and transparent account of the study being reported; that no important aspects of the study have been omitted; and that any discrepancies from the study as planned (and, if relevant, registered) have been explained.

## Data Availability

The data that support the findings of this study are not publicly available because they contain information that could compromise the privacy of research participants. De‐identified data may be made available from the corresponding author upon reasonable request and with appropriate ethical approval.
